# Using Health Information Resources for People With Cognitive Impairment (digiDEM Bayern): Registry-Based Cohort Study

**DOI:** 10.2196/54460

**Published:** 2025-01-15

**Authors:** Florian Weidinger, Nikolas Dietzel, Elmar Graessel, Hans-Ulrich Prokosch, Peter Kolominsky-Rabas

**Affiliations:** 1 Interdisciplinary Centre for Health Technology Assessment and Public Health, Friedrich-Alexander-Universität Erlangen-Nürnberg, Schwabachanlage 6, Erlangen, 91054, Germany, 49 162-2463579; 2 Department of Psychiatry and Psychotherapy, Center for Health Services Research in Medicine, Universitätsklinikum Erlangen, Friedrich-Alexander-Universität Erlangen-Nürnberg, Erlangen, Germany; 3 Department of Medical Informatics, Biometrics and Epidemiology, Friedrich-Alexander-Universität Erlangen-Nürnberg, Erlangen, Germany

**Keywords:** dementia, mild cognitive impairment, cognitive impairment, information sources, health information, health information–seeking behavior, Digital Dementia Registry Bavaria, digiDEM

## Abstract

**Background:**

Dementia is a growing global health challenge with significant economic and social implications. Underdiagnosis of dementia is prevalent due to a lack of knowledge and understanding among the general population. Enhancing dementia literacy through improved health information–seeking behavior is crucial for the self-determined management of the disease by those affected. Understanding the relationship between dementia literacy, health information–seeking behavior, and the use of various information sources among individuals with cognitive impairment is of high importance in this context.

**Objective:**

The aim of this study was to analyze the relevance of different sources of health information from the perspective of people with cognitive impairment, while also evaluating differences based on age, gender, and disease progression.

**Methods:**

This study is part of the ongoing project “Digital Dementia Registry Bavaria – digiDEM Bayern.” The Digital Dementia Registry Bavaria is a multicenter, prospective, longitudinal register study in Bavaria, Germany. People with cognitive impairment rated several information sources by using Likert scales with the values unimportant (1) to very important (5). Data were analyzed descriptively, and multiple 2-sample, 2-tailed *t* tests were used to evaluate differences by cognitive status and gender and using multiple one-way ANOVA to evaluate differences by age group.

**Results:**

Data of 924 people with cognitive impairment (531 with dementia, 393 with mild cognitive impairment) were evaluated. The most relevant health information sources were “Personal visit to a medical professional” (mean 3.9, SD 1.1) and “Family / Friends” (mean 3.9, SD 1.2). “Internet” was 1 of the 2 lowest-rated information sources by people with cognitive impairment (mean 1.6, SD 1.1), with nearly three-quarters (684/924, 74%) of the participants rating the source as unimportant. The age-specific analyses showed significant differences for the sources “Internet” (*F*_2,921_=61.23; *P*<.001), “Courses / Lectures” (*F*_2,921_=18.88; *P*<.001), and “Family / Friends” (*F*_2,921_=6.27; *P*=.002) for the 3 defined age groups. There were several significant differences between people with mild cognitive impairment and dementia whereby the first group evaluated most sources higher, such as “Internet” (mean difference=0.6; *t*_640_=7.52; *P*<.001). The only sources rated higher by the dementia group were “TV / Radio” and “Family / Friends,” with none of them showing significant differences. Gender-specific analyses showed women with cognitive impairment valuing every evaluated source higher than men apart from “Internet” (mean difference=0.4; *t*_685_=4.97; *P*<.001).

**Conclusions:**

To enhance health and dementia literacy, the best way to communicate health information to people with cognitive impairment is through interpersonal contact with medical professionals and their friends and family. Slight changes in valuation should be considered as the medical condition progresses, along with variations by age and gender. In particular, the evaluation and use of the internet are dependent on these factors. Further research is needed to capture potential changes in the valuation of the internet as a health information source.

## Introduction

Dementia is one of the most significant public health challenges of our time, and it will continue to be so in the future. To date, there are an estimated 57.4 million people with dementia worldwide, with estimates of 78 million people affected by 2030 and up to 152.8 million cases by 2050 [1,2]. In financial terms, these figures translate into an estimated global cost of dementia of US $1313.4 billion, which equates to an annual cost of US $23,796 per person with dementia [3]. Estimations also show that 75% of people with dementia worldwide live without a confirmed diagnosis. One of the reasons for this underdiagnosis is a lack of knowledge and understanding of dementia and its symptoms [1].

One way to address this problem of underdiagnosis of dementia is to improve health literacy and, therefore, dementia literacy [4]. However, levels of health literacy are low across Europe, particularly in Germany. According to the European Health Literacy Population Survey 2019‐2021, 46% of the European population and 72% of the German population had limited health literacy in 2021. These figures were even more pronounced for vulnerable subpopulations such as people of advanced age or people with chronic diseases [5].

Nutbeam [6] defines health literacy as “the ability of individuals to gain access to, understand, and use information to promote and maintain good health.” Dementia literacy is a subcategory and describes the knowledge for the recognition, management, and prevention of dementia [7]. There are multiple conceptual frameworks that describe the model of health literacy with most of them recognizing the process of using information as an essential element of health literacy. One example is the integrated model of health literacy by Sørensen et al [8], which is based on the 4 competencies of access, understanding, appraisal, and application. The first competency includes the ability to seek, find, and obtain health information. This implies that health information–seeking behavior also plays an important role in the process of how health literacy affects health outcomes [9].

Zimmerman et al [10] describe health information–seeking behavior as the purposeful behavior by an individual to find health information. The strategies for finding health information can take different forms, for example, through active seeking or more passive measures. An important aspect of health information–seeking behavior is which sources people use to obtain health information [10]. This implies a relationship between health literacy and the use of health information sources [9].

According to a recent survey conducted in 2021, 55% of Europeans aged 16‐74 years have sought health-related information via the web [11]. The US Health Information National Trends Survey indicates a similar situation for the American general population, with 72.7% using the internet first for their most recent search for health information in 2019 [12]. However, health information–seeking behavior and preferences for information sources depend heavily on various individual aspects, such as gender and age, as well as situational aspects, such as having a chronic disease like dementia [13,14]. Women are generally more interested in health information than men and also use health information sources more frequently [15]. For chronic conditions, Oh and Cho [16] found that chronic patients were generally more likely to search for health information than healthy individuals. To date, no studies have investigated which sources of health information people with cognitive impairment use and how they value them.

Therefore, the following research questions (RQs) were asked: RQ1 (How do people with cognitive impairment evaluate different sources of health information?), RQ2 (Are there differences in the evaluation based on the age of the person affected?), RQ3 (Are there differences in the evaluation based on the gender of the person affected?), and RQ4 (Are there differences in the evaluation based on the progress of the medical condition of the person affected?).

## Methods

### Study Design

This study is part of the ongoing project “Digital Dementia Registry Bavaria – digiDEM Bayern.” The Digital Dementia Registry Bavaria is a multicenter, prospective, longitudinal register study conducted in all administrative regions of Bavaria. The detailed methodology of the project is described elsewhere [17,18].

### Study Population

Participants are people with mild cognitive impairment (MCI) and people with mild or moderate dementia living in Bavaria, who are in the following collectively referred to as people with cognitive impairment. MCI is a memory impairment that results in below the expected performance for the patient’s age and level of education. People with MCI are not demented and generally maintain their independence in functional abilities of daily life [19,20]. To identify eligible participants, people have to undergo a screening based on the Mini-Mental State Examination (MMSE) and Montreal Cognitive Assessment (MoCA) prior to inclusion [21,22]. If available, a family caregiver is included with the person with cognitive impairment [17,18].

### Recruitment and Data Collection

Participants are recruited by specially trained research partners in all of the 7 administrative regions in Bavaria, beginning in August 2020. Research partners are institutions that are specialized and have experience in the management and care of people with cognitive impairment and their family caregivers. Data collection is conducted through standardized face-to-face interviews using a web-based data entry system [17,18].

### Ethical Considerations

The Digital Dementia Registry Bavaria is performed following the ethical standards of the Declaration of Helsinki, and it obtained ethical approval from the ethics committee of the Medical Faculty of the Friedrich-Alexander-Universität Erlangen-Nürnberg (application number: 253_20 B). Informed consent from the participants or their authorized representative is acquired before screening and study inclusion. The project collects and stores all personal data separately from the registry data on different stand-alone systems to ensure data protection. All participants are pseudonymized. The data protection concept was approved by the local data protection supervisor of the Friedrich-Alexander-Universität Erlangen-Nürnberg and authorized by the Bavarian Data Protection Commissioner. Participation is voluntary, and participants are not compensated [17].

### Measures

Sociodemographic data of the people with cognitive impairment were collected, such as age, gender, educational degree, family status, score in cognitive assessment, and whether they had a family caregiver.

The relevance of 8 different sources of health information was rated by people with cognitive impairment using Likert scales ranging from unimportant (1) to very important (5). Based on previous studies, the rated health information sources were “Internet,” “TV / Radio,” “Books / Brochures,” “Courses / Lectures,” “Newspaper / Journals,” “Family / Friends,” “Pharmacy,” and “Personal visit to a medical professional” [23,24]. In order to further evaluate the target group’s use of the internet, the participants were asked about the last time they had used the internet. Participants who had last used the internet 3 months ago or had never used it were also asked about their reasons for not using it.

### Statistical Analysis

Subgroup analyses were performed for the cognitive status and gender of the people with cognitive impairment using multiple 2-sample, 2-tailed *t* tests and for the age of the participants using multiple one-way ANOVA to evaluate potential differences depending on the progress of the medical condition and gender- and age-specific differences. Based on the age distribution in the sample, the participants were divided into 3 age groups (<70 y, 70‐79 y, and 80 y or older) for the analysis of age-specific differences. In order to minimize or avoid the probability of a type 1 error, we applied the Bonferroni correction for multiple testing. Accordingly, the adjusted significance level of *P*<.002 was set for all subgroup analyses (nonadjusted significance level: *P*<.05) [25]. Effect sizes (Cohen *d* or η²) were determined [26]. Statistical analysis was performed using IBM SPSS Statistics (version 28; IBM Corp).

## Results

### Study Population

In the period between August 2020 and July 2023, in total, 924 people with cognitive impairment were included in the Digital Dementia Registry Bavaria. The following presented analyses refer to the baseline survey.

Table 1 shows the characteristics of the participants in total and for the 2 subgroups “MCI” and “dementia.” The age of the study population ranged from 54 to 102 years and was 80.5 (SD 7.7) years on average. Around 59.4% (549/924) of the participants were female, with a mean MMSE score of 22.8 (SD 3.8) points and a mean MoCA result of 19.9 (SD 2.7) points. With 58.2% (538/924), most participants had a middle education, and 50.3% (465/924) were in a partnership. Around 64.1% (592/924) of the people with cognitive impairment regularly received help from a family caregiver at least once per week. Based on their MMSE score, 42.5% (393/924) of the study population was assigned to the MCI group and 57.5% (531/924) of the study population was assigned to the dementia group. There were significant differences between the 2 subgroups for their age (*P*<.001), their MMSE result (*P*<.001), their education (*P*<.001), their family status (*P*=.003), and whether they regularly received support from a family caregiver (*P*<.001).

**Table 1.** Description of this study population with the classification into the groups “MCI” (mild cognitive impairment) and “dementia” in a registry-based cohort study in Germany from 2020 to 2023.

		Total (N=924)	MCI (n=393; 42.5%)	Dementia (n=531; 57.5%)	*P* value
Age (years), mean (SD)		80.5 (7.7)	78.3 (7.8)	82.1 (7.2)	<.001
Gender, n (%)					.46
	Female	549 (59.4)	228 (58.0)	321 (60.5)	
	Male	375 (40.6)	165 (42.0)	210 (39.5)	
Family caregiver, n (%)					<.001
	No family caregiver	332 (35.9)	201 (51.1)	131 (24.7)	
	Has family caregiver	592 (64.1)	192 (48.9)	400 (75.3)	
Cognitive assessment, mean (SD)					
	MMSE^a^	22.8 (3.8)	26.5 (1.7)	20.1 (2.5)	<.001
	MoCA^b^	19.9 (2.7)	19.9 (2.7)	—^c^	—
Education, n (%)					<.001
	Lower education	197 (21.3)	62 (15.8)	135 (25.4)	
	Middle education	538 (58.2)	234 (59.5)	304 (57.3)	
	Higher education	189 (20.5)	97 (24.7)	92 (17.3)	
Family status, n (%)					.003
	No partnership	459 (49.7)	173 (44.0)	286 (53.9)	
	In partnership	465 (50.3)	220 (56.0)	245 (46.1)	

a MMSE: Mini-Mental State Examination.

b MoCA: Montreal Cognitive Assessment.

c Not applicable.

### General Valuation of Health Information Sources

The most relevant health information sources for people with cognitive impairment were “Family / Friends” as well as “Personal visit to a medical professional” (eg, doctors or physiotherapists) (Table 2). With an average score of 3.9 (SD 1.1), 33.0% (305/924) of the people with cognitive impairment rated “Personal visit to a medical professional” as a very important source, and around three-quarters (694/924, 75.1%) rated it as at least “important.” A similar result was found for “Family / Friends,” with an average score of 3.9 (SD 1.2), and 39.6% (366/924) of the people with cognitive impairment rated them as a very important source of health information. Going by the highest average score, these 2 sources were followed by “TV / Radio,” “Newspaper / Journals,” “Pharmacy,” and “Books / Brochures.” The 2 lowest-rated information sources by people with cognitive impairment were “Internet” and “Courses / Lectures,” with an average score of 1.6 (SD 1.1) and 1.6 (SD 1.0) on the Likert scale. Nearly three-quarters (684/924, 74.0%) of the participants rated “Internet” as an unimportant source, with a similar amount (680/924, 73.6%) stating that they had not used the internet in the last 3 months; the majority of those participants (569/680, 83.7%) stated that they had never used the internet before. The most frequently cited reasons for not using the internet were “generally no interest in the internet” (446/680, 65.6%) and “too complicated” (386/680, 56.8%) (Multimedia Appendix 1).

**Table 2.** General evaluation of health information sources by people with cognitive impairment using Likert scales ranging from unimportant (1) to very important (5) in a registry-based cohort study in Germany from 2020 to 2023.

	Likert scale ratings (% of participants who selected each rating)					Likert scale score, mean (SD)
	Unimportant	Less important	Partially	Important	Very important	
Internet	74.0	6.8	8.0	7.4	3.8	1.6 (1.1)
TV/Radio	13.7	9.3	20.9	38.5	17.5	3.4 (1.3)
Books/Brochures	41.0	19.9	18.1	14.0	7.0	2.3 (1.3)
Courses/Lectures	69.0	13.6	9.4	6.5	1.4	1.6 (1.0)
Newspaper/Journals	18.8	11.9	19.4	33.7	16.2	3.2 (1.4)
Family/Friends	8.0	7.3	13.2	31.9	39.6	3.9 (1.2)
Pharmacy	26.8	12.3	19.7	28.6	12.6	2.9 (1.4)
Personal visit to a medical professional	6.6	5.7	12.6	42.1	33.0	3.9 (1.1)

### Valuation of Health Information Sources by Age

The age-specific analysis shows heterogeneous results for the 8 health information sources (Multimedia Appendix 2). The sources “Internet” (*F*_2,921_=61.23; *P*<.001), “Courses / Lectures” (*F*_2,921_=18.88; *P*<.001), and “Family / Friends” (*F*_2,921_=6.27; *P*=.002) showed significant differences for the 3 defined age groups. People with cognitive impairment valued “Internet” and “Courses / Lectures” lower as age increased, from people under the age of 70 years (“Internet”: mean 2.7, SD 1.5; “Courses / Lectures”: mean 2.1, SD 1.3), to people aged 70 to 79 years (“Internet”: mean 1.8, SD 1.3; “Courses / Lectures”: mean 1.7, SD 1.0), and to people aged 80 years and older (“Internet”: mean 1.3, SD 0.9; “Courses / Lectures”: mean 1.4, SD 0.9). In comparison, the valuation of the information source “Family / Friends” grew with increasing age, from people younger than 70 years (mean 3.7, SD 1.2) and people between the ages of 70 and 79 years (mean 3.7, SD 1.3), to people aged 80 years and older (mean 4.0, SD 1.2). While age-specific differences in the information sources “Family / Friends” (*η²*=0.01) and “Courses / Lectures” (*η²*=0.04) showed small effect sizes, the effect size for the information source “Internet” (*η²*=0.12) was medium.

### Valuation of Health Information Sources by Gender

Concerning the gender-specific evaluation of information sources, the results show that apart from “Internet” and “Personal visit to a medical professional,” women with cognitive impairment valued every explored source more than men with cognitive impairment on average (Multimedia Appendix 3). Two of the information sources showed significant gender-specific differences. As already described, men with cognitive impairment rated “Internet” 0.4 points higher on average than women with cognitive impairment (*t*_685_=4.97; *P*<.001), with a small effect size (Cohen *d*=0.35). Female participants valued “Pharmacy” higher than male participants by 0.4 points on average (*t*_772_=3.98; *P*<.001), with a small effect size (Cohen *d*=0.27). Another information source that was valued higher by women with cognitive impairment was the source “Family / Friends,” with a mean difference of 0.2 (*t*_756_=2.49; *P*=.013) and a small effect size (Cohen *d*=0.17).

### Valuation of Health Information Sources by Cognition

Regarding the relevance of health information sources categorized by cognitive status, there were several significant differences between the MCI and dementia groups (Multimedia Appendix 4). People with MCI rated the sources “Internet,” “Books / Brochures,” “Courses / Lectures,” “Newspapers / Journals,” “Pharmacy,” and “Personal visit to a medical professional” higher. Except for the sources “Books / Brochures” and “Newspapers / Journals,” all of those group differences showed significant results. The source “Internet” showed the most considerable mean difference between both groups, with 0.6 points (*t*_640_=7.52; *P*<.001) and a medium effect size (Cohen *d*=0.53). The only sources rated higher by the dementia group were “TV / Radio” and “Family / Friends,” with none of them showing significant differences.

## Discussion

### Principal Results

To the best of our knowledge, this is the first study that describes the relevance of different sources of health information from the perspective of people with cognitive impairment.

Our analyses show that interpersonal contact with family and friends and that with medical professionals are the most important sources of health information for people with cognitive impairment. This interpersonal communication is followed closely by traditional media formats such as TV, radio, newspapers, and journals. People with cognitive impairment rated the internet and attending courses or lectures the lowest, with each being valued as less important or unimportant.

### General Valuation of Health Information Sources

There are a number of possible reasons for the low relevance of the internet for people with cognitive impairment. The participants of our study mainly reported that they were generally not interested in the internet as a medium and that the internet was too complicated for them. Other frequently cited reasons were that relatives take care of matters on the internet, no advantage is seen in using it, or traditional media are sufficient. These findings are partially in line with the literature, although it should be noted that the majority of the participants in our study had never used the internet before and therefore had no experience with the internet. Dixon et al [27] defined 4 main barriers that prevented people with cognitive impairment from seeking the internet for health information. These are the scarcity of relevant information and the inaccessibility, inaccuracy, and distrust of the information that was found [27]. More importantly, Dixon et al [28] see a chance of the internet becoming a more relevant source for people with cognitive impairment in the future, especially if their close relatives and health professionals support them in validating internet-based health information.

Our findings are in line with the large-scale survey by Weber et al [29] who assessed adults older than 60 years and reported high use of interpersonal communication with medical professionals, family, and friends as well as traditional mass media or brochures and infrequent use of the internet for seeking health information.

Stehr et al [30] came to similar results and showed that interpersonal contact and traditional mass media are the most used health information sources for people aged 65 years or older while they also confirmed that the internet is used little or not at all. Further, they noticed that the use and valuation of communication with medical professionals increases for people with chronic diseases, which include cognitive impairment [30].

The above results can be explained by, among other things, the trust that older adults or people with cognitive impairment place in the various sources. Baumann et al [31] reported that medical professionals, in this case doctors, are the most trusted health information source followed by family and friends, while the internet is one of the least trusted sources. These findings implicate that the trustiness of the various sources is a significant factor in the valuation by people with cognitive impairment.

Pertl et al [32] also stated that people with MCI show problems with understanding numerical health information. These problems are likely to increase for people with dementia, as they found a statistically significant correlation with global cognitive status [32]. These findings suggest that people with MCI and dementia might require explanation through communicating with medical professionals and their friends or family to understand their health issues.

### Valuation of Health Information Sources by Age

The age-specific results show that the relevance of the information sources “Internet” and “Courses / Lectures” decreased significantly with age. Contrary to this, the evaluation of the source “Family / Friends” enlarged with increasing age. No significant differences were found for the 5 other sources based on participants’ age. The difference in the information source “Internet” is in particular noteworthy, with a medium effect size and high differences in the mean values between the 3 age groups.

Our age-specific findings regarding the internet align with the results of the “D80+ report” by Reissmann et al [33], which found low internet use among people older than 80 years in Germany, at 37%. Weber et al [29] also found that age is a decisive factor influencing internet use as the tendency of infrequent use of the internet for seeking health information became more pronounced with increasing age.

Reasons for these observations may include the often limited digital health literacy or generally limited digital media literacy and internet skills of the older population [34]. These limitations become even more pronounced with increasing age, especially for people with cognitive impairment [34,35]. The high average age of 80.5 years in this sample could therefore also explain the generally low rating of the internet as a source of health information.

### Valuation of Health Information Sources by Gender

Our gender-specific analyses show that women and men with cognitive impairment differ in assessing the relevance of health information sources. Except for the internet, all our evaluated sources were more important to women with cognitive impairment, though these differences between men and women were small. The sources “Pharmacy” and “Family / Friends” showed the most significant differences.

These gender-specific findings are in line with current scientific research. For the general population, Manierre [15] discovered that women seek health information more often than men. In the group of adults aged 65 years and older, Stehr et al [30] found higher use of most health information sources by women. They also noted the most remarkable differences between the sources “Pharmacy” and “Internet,” with the former being much more used by women and the latter by men [30]. The same applies to the results of the KomPaS study by Horch et al [36], who found similar differences in the use of these 2 sources.

### Valuation of Health Information Sources by Cognition

When categorized by cognitive status, our results show that people with MCI rate most of the health information sources evaluated more highly than people with dementia. People with dementia only value contact with family and friends as well as watching TV or listening to the radio more.

It is noticeable that the mean values for health information sources that require some activity to obtain information, for example, leaving home to see a doctor, are all higher for the group of people with MCI. The same applies to the mean for sources of information that can be used passively, such as watching television or receiving advice from a friend, which is higher for people with dementia. This could be explained by the progressive social withdrawal of people with cognitive impairment as the medical condition progresses [37].

Dixon et al [28] also found similar results with their research on the changing strategies of people with cognitive impairment seeking health information. They reported that most people with cognitive impairment apply an active search technique after receiving their diagnosis and then transition to a monitoring strategy for ongoing seeking of health information. With the progression of their medical condition, they transition to a proxy information search, as they have problems accessing the health information needed when just actively searching by themselves. With further progression, the authors even describe a strategy of information avoidance and selective exposure to health information by people with cognitive impairment [28]. These changes in information behavior could explain the distribution of different sources in the 2 cognitive groups. While people with MCI are likely to use one of the first, more active search strategies described, people with dementia are more likely to use one of the latter strategies due to their disease progression.

### Limitations

This study has limitations. First, our sample was a convenience sample with a specific target group of people with cognitive impairment in Bavaria. We cannot exclude that we may have a selection bias as a result of the different referral patterns in the different regions of Bavaria. Although our research partners were institutions specialized in managing and caring for people with cognitive impairment in all of the 7 administrative regions in Bavaria, this study is not representative of the total population. This limits the comparability and transferability of the results to the entirety of people with cognitive impairment.

Furthermore, the results of the group differences mostly show small effect sizes, except for the group differences between people with MCI and dementia and the 3 age groups for the information source “Internet.” This limits the power and generalizability of the results while highlighting the need for further research with even larger case numbers.

### Conclusions

To enhance health and dementia literacy in people with cognitive impairment, our results suggest that interpersonal contact with medical professionals and their friends and family is the best way to communicate health information to people with cognitive impairment. Another way is to disseminate information through traditional mass media. As the disease progresses, the importance of family and friends as sources of information increases, while the relevance of other sources decreases. Women with cognitive impairment are generally easier to reach and more interested in health information. At the same time, men and younger people with cognitive impairment also use the internet to some extent and are open to receiving information via this medium.

While the internet was of little importance as a source of health information to most of our study population of people with cognitive impairment older than 70 years, this may change in the future, as recent surveys from the European Union and the United States indicate [11,12]. Due to the continuing improvement in digital literacy in older adults, the internet might become more valuable as a source of health information. This implicates a need for further research in the future to capture these potential changes.

## Supplementary material

10.2196/54460 Multimedia Appendix 1 Frequency of reasons given for not using the internet by people with cognitive impairment in a registry-based cohort study in Germany from 2020 to 2023.

10.2196/54460 Multimedia Appendix 2 Evaluation of health information sources by people with cognitive impairment, with differentiation by age, using Likert scales ranging from unimportant (1) to very important (5) in a registry-based cohort study in Germany from 2020 to 2023.

10.2196/54460 Multimedia Appendix 3 Evaluation of health information sources by people with cognitive impairment, with differentiation by gender, using Likert scales ranging from unimportant (1) to very important (5) in a registry-based cohort study in Germany from 2020 to 2023.

10.2196/54460 Multimedia Appendix 4 Evaluation of health information sources by people with cognitive impairment, with differentiation by their cognitive status, using Likert scales ranging from unimportant (1) to very important (5) in a registry-based cohort study in Germany from 2020 to 2023.

## Abbreviations

MCI: mild cognitive impairment
MMSE: Mini-Mental State Examination
MoCA: Montreal Cognitive Assessment
RQ: research question
