# Supplementary figures and images for: Using Health Information Resources for People With Cognitive Impairment (digiDEM Bayern): Registry-Based Cohort Study
Source: JMIR Form Res. 2025 Jan 15;9:e54460. doi: 10.2196/54460 (PMC11769685; doi:10.2196/54460)

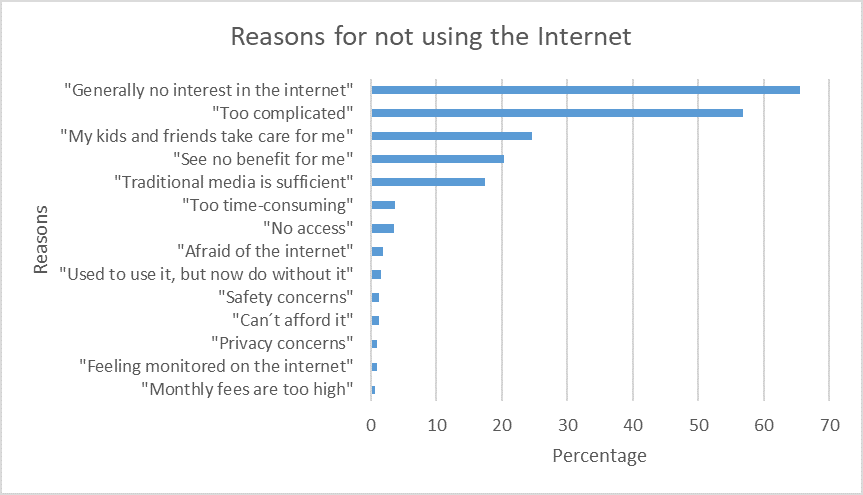

Supplement: Multimedia Appendix 1 [file formative-v9-e54460-s001.png]

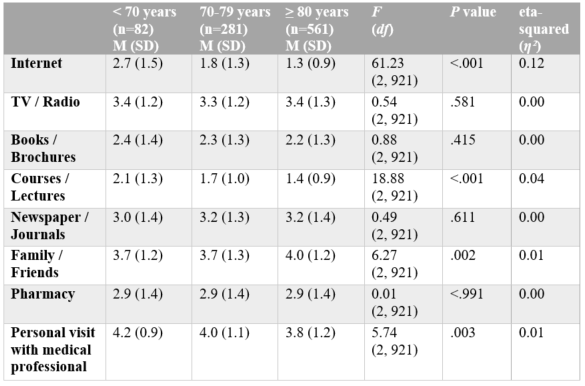

Supplement: Multimedia Appendix 2 [file formative-v9-e54460-s002.png]

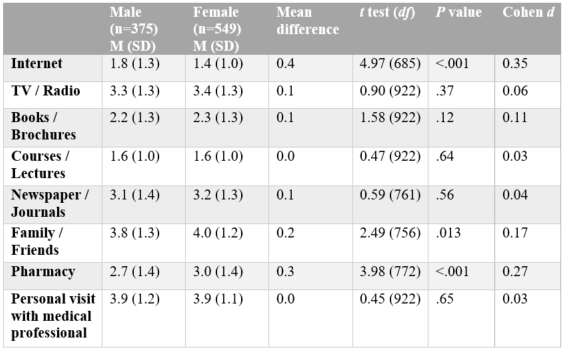

Supplement: Multimedia Appendix 3 [file formative-v9-e54460-s003.png]

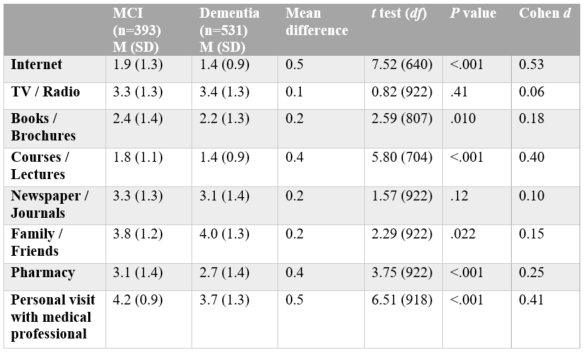

Supplement: Multimedia Appendix 4 [file formative-v9-e54460-s004.png]
